# Supplementary material for: DNA processing by the Kaposi's sarcoma-associated herpesvirus alkaline exonuclease SOX contributes to viral gene expression and infectious virion production
Source: Nucleic Acids Res. 2022 Dec 20;51(1):182–97. doi: 10.1093/nar/gkac1190 (PMC9841436; doi:10.1093/nar/gkac1190)
Supplement: gkac1190_Supplemental_File [file gkac1190_supplemental_file.pdf]

Supplementary Figure 1

A

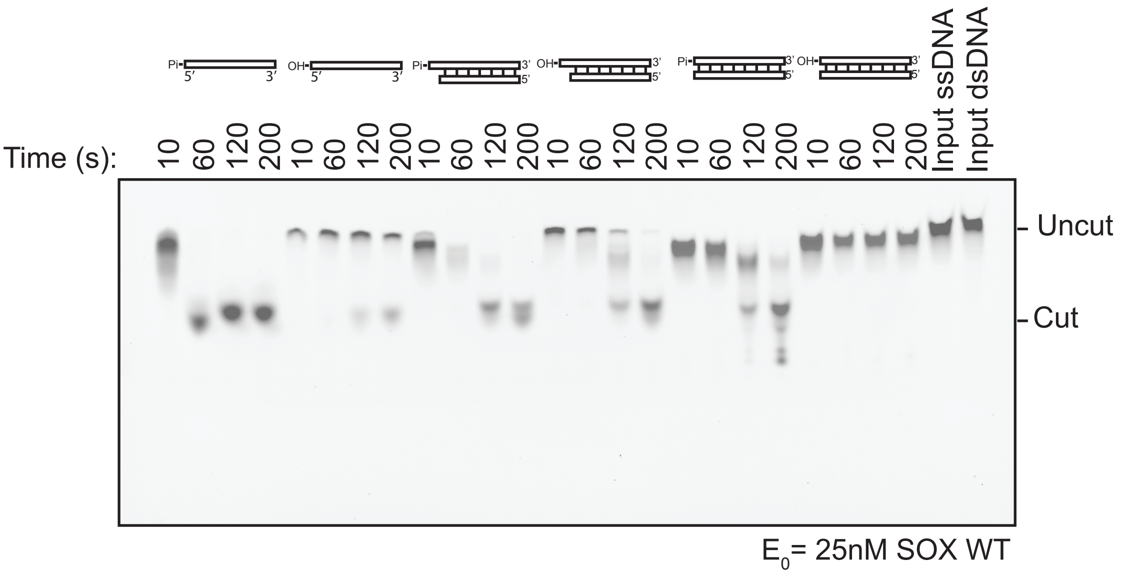

B

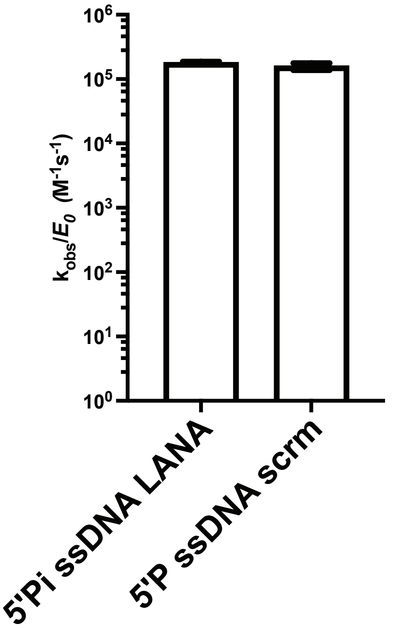

C

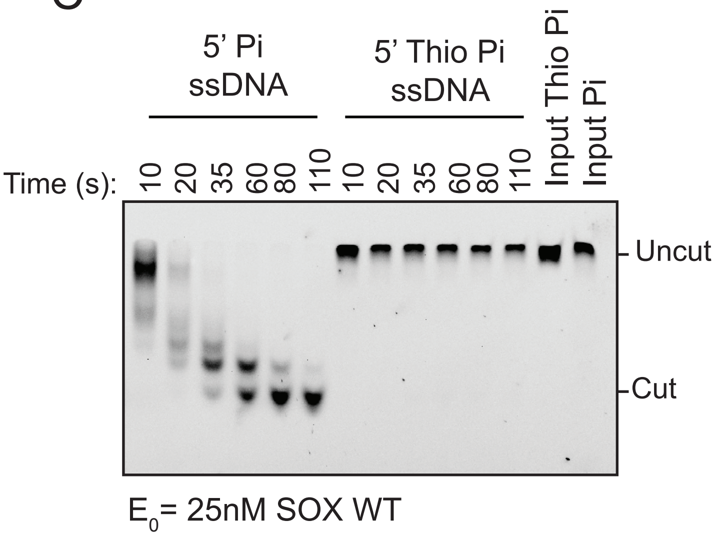

(A) Urea-PAGE gel showing DNA processing by WT SOX of the dsDNA and ssDNA substrates used in Figure 1B. DNA substrates contain either a 5' phosphate (Pi) or hydroxyl (OH) group. All substrates contain a 3' fluorophore, which is located on the antisense strand for dsDNA substrates. Reactions were carried out at concentration of 25 nM SOX and 1 nM of DNA substrate. Increased time is indicated by the black bar. The time (seconds) are listed above the gel indicate points at which the reactions were stopped. (B) The catalytic efficiency of SOX processing of a 25 bp ssDNA LANA and a 25 bp ssDNA scrambled sequence. Reactions were run at a constant concentration of 25 nM and 1 nM DNA substrates. Reactions were run in triplicate and in substrate limiting conditions. (C) Urea-PAGE gel showing WT SOX (25 nM) processing of a 5' phosphate containing DNA probe under substrate (1 nM) limiting conditions, and inhibition of processing by the 5' thio-phosphate DNA probe. The time (seconds) are listed above the gel indicate points at which the reactions were stopped. Markers indicate cut and uncut DNA products.

Supplementary Figure 2

A

```
KSHV ORF37    101 ASVYAACSQMNAHQRHHICCLVERATSSQSLNPVWDALRDGIISSSKFHWAVKQQNTSKK
MHV68 ORF37    99 SAVYAACRDVTPDIKYAVCQRIEALTRGQQDNELWDILRDGMISSSKFQWAVKQHSINKK
EBV BGLF5      85 KSIYWGLQEATDEQRTVLCSYVESMTRGQSENLMWDILRNGIISSSKLLSTIKNG--PTK
HSV1 UL12     204 -------AAADAVAPRPLMGFYEAATQNQADCQLWALLRRGLTTASTLRWGPQGPCFSPQ
VZV ORF48     106 RPEELA-SKLTQDDIKRILLTIESETRGQGDNAIWTLLRRNLITASTLKWSVSGPVIPPQ
EXO LAMBD       9 --------------RT----GIDVRAVEQGD-DAWHKLRLGVITASEVHNVIAKPRSGKK

KSHV ORF37    161 IFSPWP------------ITNNHFVAGPLAFGLRCEEVVKTLLATLLHPDE---------
MHV68 ORF37   159 LFNPQP------------IKVNHYFAGPLAFGIRCEQTVKKILSELIHPNL---------
EBV BGLF5     143 VFEPAP------------ISTNHYFGGPVAFGLRCEDTVKDIVCKLICGDA---------
HSV1 UL12     257 WLKHNA------------SLRPDVQSSAVMFGRVNEPTARSLLFRYCVGRADD----GGE
VZV ORF48     165 WFYHHN------------TTDTYGDAAAMAFGKTNEPAARAIVEALFIDPADIRTPDHLT
EXO LAMBD      50 WPDMKMSYFHTLLAEVCTGVAPEVNAKALAWGKQYENDARTLFEF---------------

KSHV ORF37    200 --------------------------ANCLDYGFMQSPQNGIFGVSLDFAANVKTDTEGR
MHV68 ORF37   198 --------------------------PRFHDCGFLPSAIDGIFGVSLDTAFNVFTDSSGL
EBV BGLF5     182 --------------------------SANRQFGFMISPTDGIFGVSLDLCVNVESQ-GDF
HSV1 UL12     301 AGADTRRFIF-----HEPSDL---AEENVHTCGVLMDGHTGMVGASLDILVCPRD-IHGY
VZV ORF48     213 PEATTKFFNFDMLNTKSPSLLVGTPRIGTYECGLLIDVRTGLIGASLDVLVCDRDPLTGT
EXO LAMBD      95 -----------------------TSGVNVTESPIIYRDESMRTACSPDGLC---------

KSHV ORF37    234 LQFDP------NCKVYEIKCRFKY----TFAKME-CDPIYAAYQRLYEAPGKLALKDFFY
MHV68 ORF37   232 VHFEP------DSIVYEIKSRYKY----QFSKSE-FDGLAKKYLDLYKNPCEATFIKFIN
EBV BGLF5     215 ILFTD------RSCIYEIKCRFKY----LFSKSE-FDPIYPSYTALYKRPCKRSFIRFIN
HSV1 UL12     352 LAPVP-KT---PLAFYEVKCRAKY----AFDPMDPSDPTASAYEDLMAHRSPEAFRAFIR
VZV ORF48     273 LNPHPAET---DISFFEIKCRAKY----LFDPDDKNNPLGRTYTTLINRPTMANLRDFLY
EXO LAMBD     114 ----------SDGNGLELKCPFTSRDFMKFRLGG-------------------------

KSHV ORF37    283 SISKPAVEYVGLGKLPSESDYLVAYDQEWEACPRK--KRKLTPLHNLIRECILHNSTTES
MHV68 ORF37   281 CISRPAVEYVPHGKLPSESDYLITHSMNWKTSNKR--KRKITDSHICLKKCLLHNMYQQS
EBV BGLF5     264 SIARPTVEYVPDGRLPSEGDYLLTQDEAWNLKDVR--KRKLGPGHDLVADSLAANRGVES
HSV1 UL12     404 SIPKPSVRYFAPGRVPGPEEALVTQDQAWSEAHASGEKRRCSAA---DRALVELNSGVVS
VZV ORF48     326 TIKNPCVSFFGPSANPSTREALITDHVEWKRLGFK-GGRALTEL---DAHHLGLNRTISS
EXO LAMBD     138 ------------------------------------------------------------

KSHV ORF37    341 DVYVLTDPQDTRGQISIKAR------FKANLFVNVRHSYFYQVLLQSSIVEEYIGLD-SG
MHV68 ORF37   339 TVYILSDPSETSGKITIKAS------FPIDVFVNPAHNYFYQVALQNMVVQDYIEFG-QG
EBV BGLF5     322 MLYVMTDPSENAGRIGIKDR------VPVNIFINPRHNYFYQVLLQYKIVGDYVRHSGGG
HSV1 UL12     461 EVLLFGAPDLGRHTISPVSWSSGDLVRREPVFANPRHPNFKQILVQGYVLDSHFP-----
VZV ORF48     382 RVWVFNDPDIQKGTITTIAWATGDTALQIPVFANPRHANFKQIAVQTYVLSGYFP-----
EXO LAMBD     138 -------------------------------FEAIKSAYMAQVQYSMWVTRKNAWYFA--
```

Conserved

Active site

DNA Binding

B

SOX catalytic and conserved residues

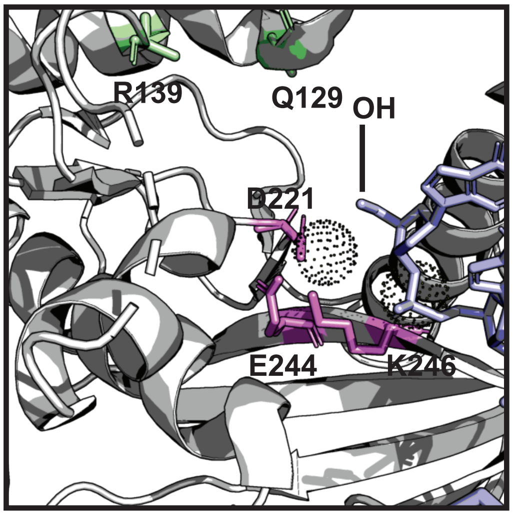

C

SOX putative DNA binding residues

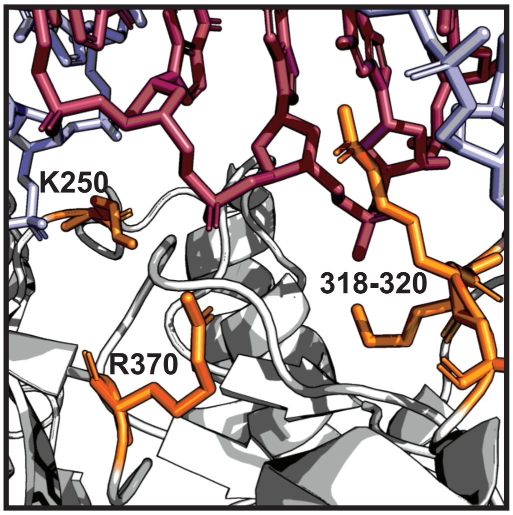

(A) Sequence alignment using Expasy uiprot alignment tool of herpesviral exonucleases spanning alpha- and gamma-herpesvirus subfamilies as well as λ exonuclease. Conserved residues are highlighted in green. Residues highlighted in orange contact DNA in PDB:3POV structure. Residues highlighted in pink indicate residues predicted to be part of the active site. (B) Structure of the KSHV SOX active site. Catalytic residues are highlighted pink, conserved residues highlighted in green and spotted spheres are magnesium site 1 and site 2. The 5' hydroxyl DNA is colored light blue and positioned in the active site (PDB:3POV). (C) Putative DNA binding residues are highlighted in orange. Double stranded DNA (light blue 5' and cyan 3') is modeled into SOX (36).

Supplementary Figure 3

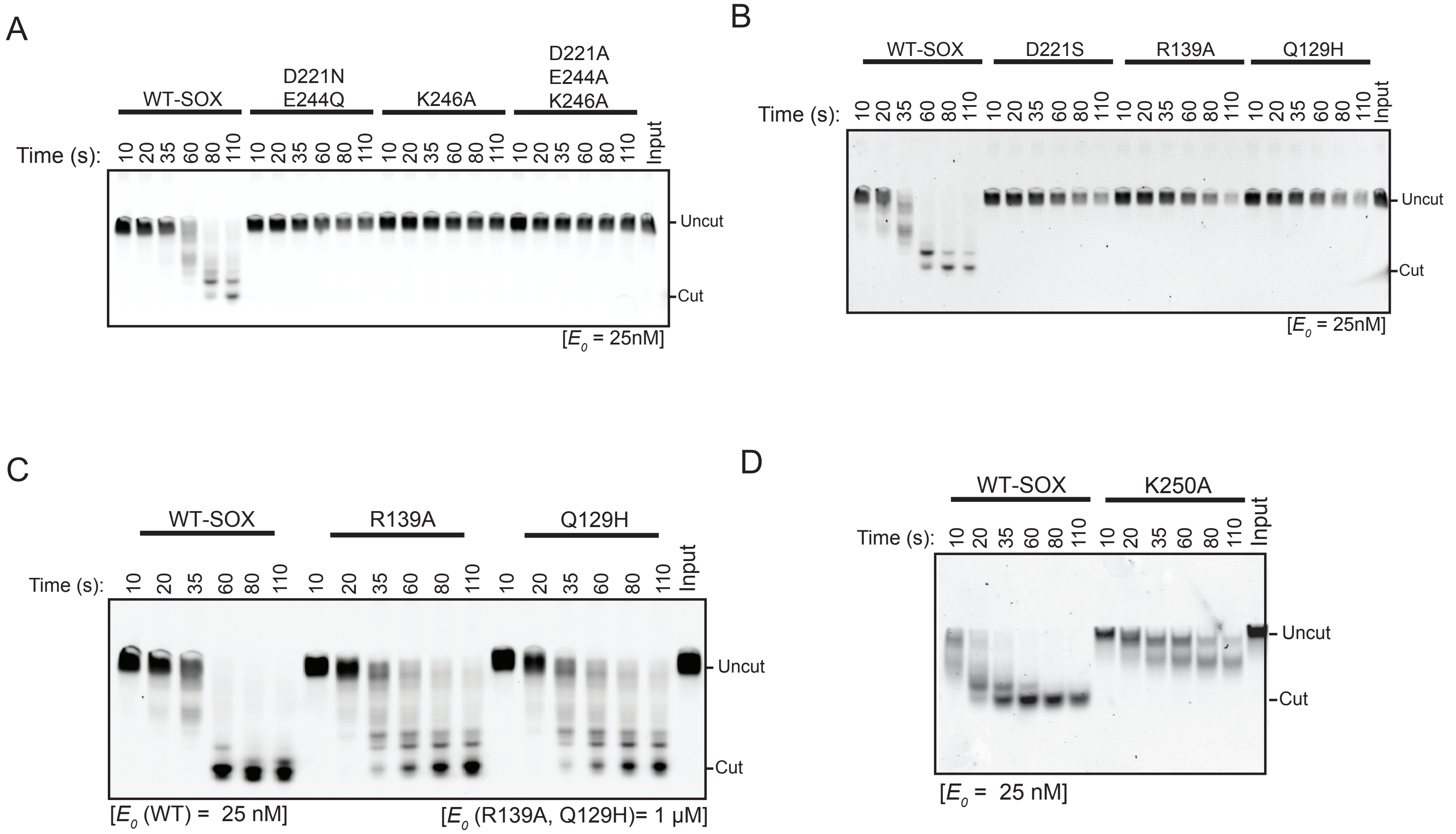

(A) Urea-PAGE gel showing DNA processing by WT SOX and the D221N/E224Q, K246A and D221A/E244A/K246A (3X) SOX mutants. The reactions were run at 25 nM of enzyme and 5 nM DNA substrate. Hash marks denote uncut and cut DNA substrate. The time (seconds) are listed above the gel and indicate points at which the reactions were stopped. (B) Urea-PAGE gel showing DNA processing by WT, D221S, R139A and Q129H SOX. The reactions were run at 25 nM of enzyme and 1 nM DNA substrate. Hash marks denote uncut and cut DNA substrates. The time (seconds) are listed above the gel and indicate points at which the reactions were stopped. (C) Urea-PAGE gel showing processing defects of SOX mutants in conserved residues R139A and Q129H. The reactions were run at 1 μM of R139A and Q129H, and 25 nM of WT SOX enzyme and 1 nM DNA substrate. Hash marks denote uncut and cut DNA substrates.The time (seconds) are listed above the gel and indicate points at which the reactions were stopped. (D) Urea-PAGE gel showing DNA processing by K250A (25 nM), WT SOX (25 nM) and 1 nM DNA substrates. Hashmarks denote uncut and cut DNA substrates. The time (seconds) are listed above the gel and indicate points at which the reactions were stopped.

Supplementary Figure 4

A

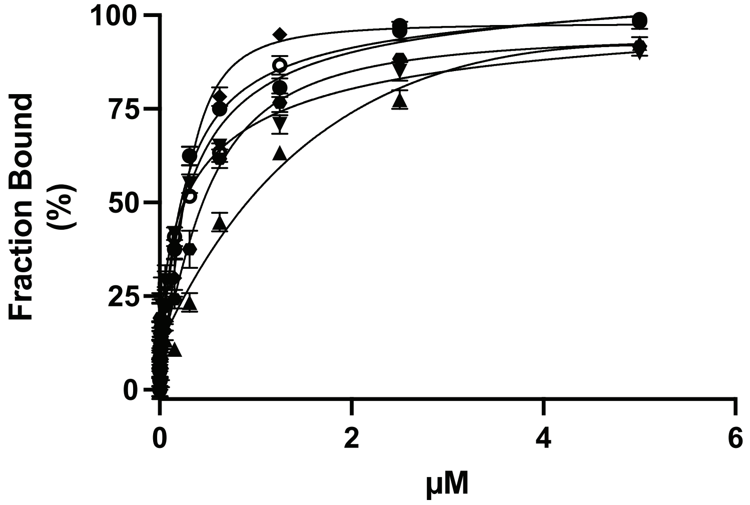

SOX	Affinity (nM)
WT	143 ± 33
Q129H	105 ± 40
K250A	649 ± 39
R139A	189 ± 49
R370A	120 ± 45
318-320A	120 ± 39

(A) Equilibrium binding measurements of WT SOX (hollow circle), Q129H (solid circle) K250A (triangle), R139A (diamond), R370A (hexagon), 318-320A (upside down triangle). A constant concentration (1nM) of a fluorescently labeled LANA probe was used as described in Figure 1A. Data represent 3 biological replicates. The table shows calculated Kd values for SOX proteins using a signal binding model.

Supplementary Figure 5

A

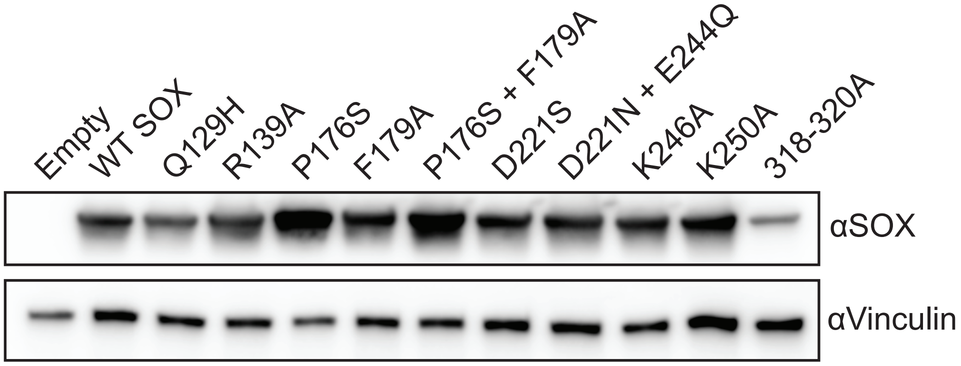

A) Western blot of 293T cell lysates transiently transfected with the indicated SOX mutant expression constructs and probed with an anti-SOX antibody. Vinculin serves as a loading control.

Supplementary Figure 6

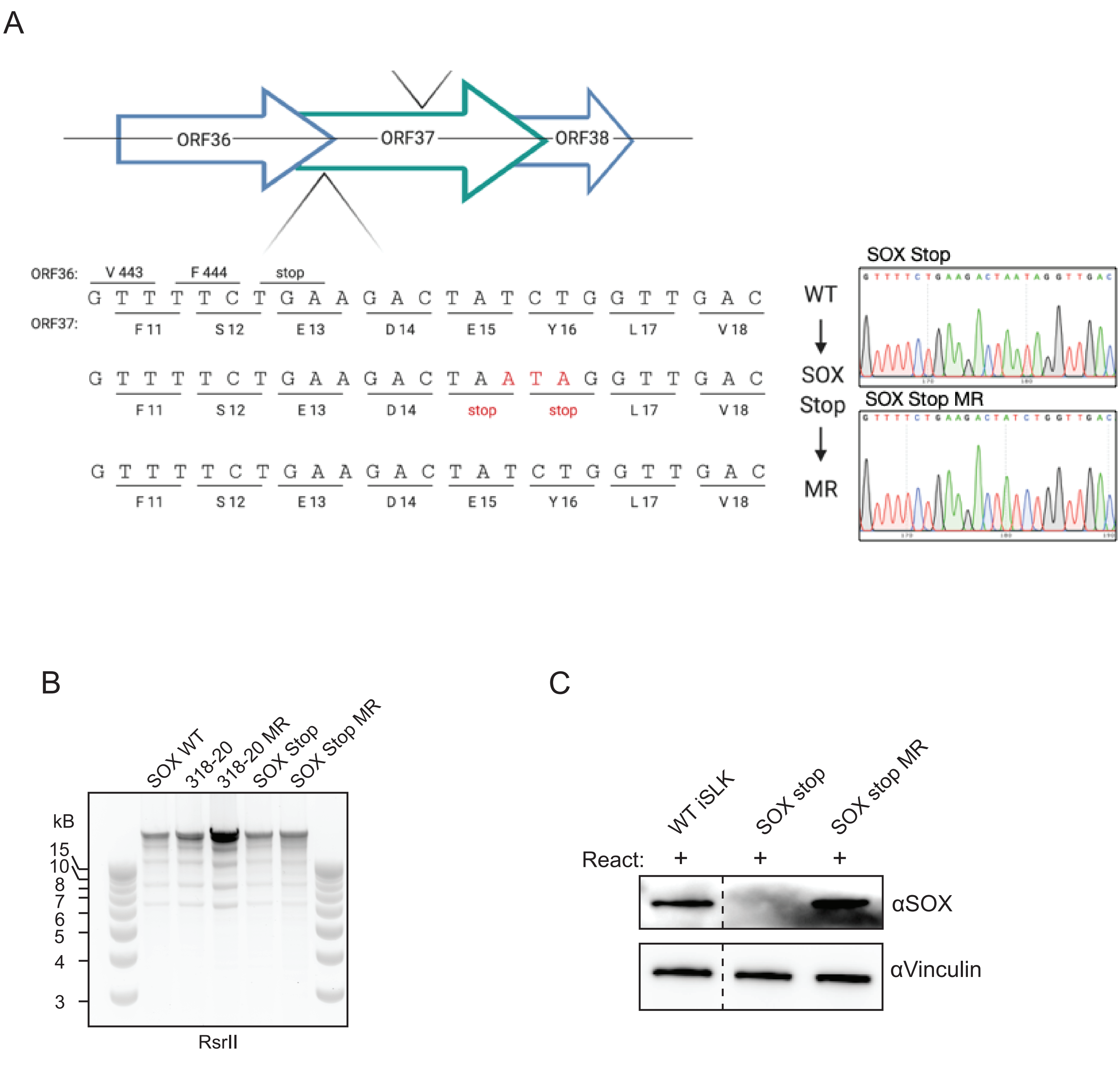

A) Diagram depicting the genomic locus of the engineered SOX Stop mutant, shown in red. Sanger sequencing for SOX Stop and the MR cell line at the site of mutation is shown adjacent to the diagram. (B) RsrII digestions of the engineered BACs in comparison to the WT Bac16 to demonstrate no large-scale rearrangements in BAC DNA. (C) Western blot of whole cell lysates from WT iSLK cells, the KSHV mutant that does not express SOX (SOX stop) and a mutant rescue version of KSHV where SOX expression is restored (SOX stop MR). Lysates were harvested 72 hours post lytic reactivation and blotted with an anti-SOX antibody. Vinculin serves as a loading control. Blot was cropped (dashed lines) to omit two lanes containing other samples between WT iSLK and SOX Stop for simplicity.

Supplementary Figure 7

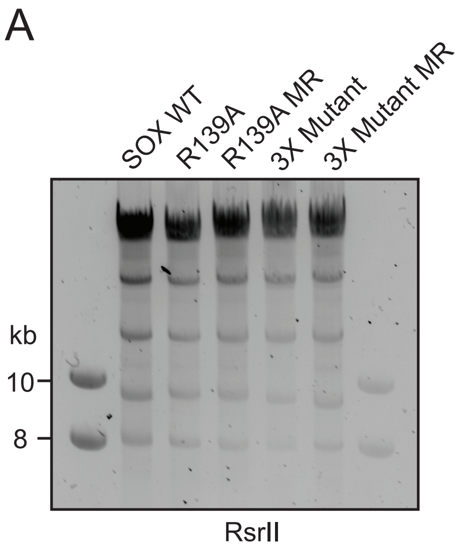

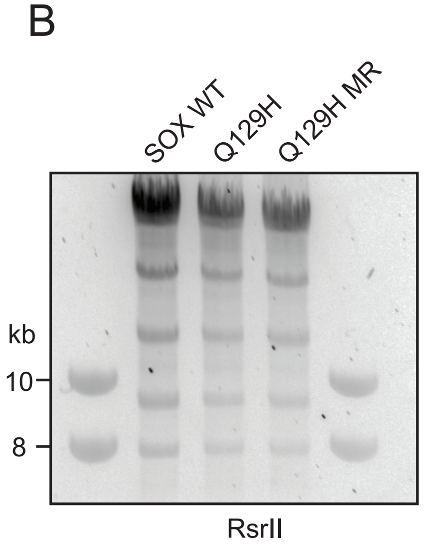

(A,B) RsrII digestions of the engineered BACs in comparison to the WT Bac16 to demonstrate no large-scale rearrangements in BAC DNA.

Supplementary table 1

mutant	function	ssDNA catalytic efficiency M^{-1}/s^{-1}	reference
WT SOX		$1.78x10^6$ +/- $4.3x10^3$	this paper
P176S*	RNA processing	N/A	(25)
F179A*	RNA processing	N/A	(25)
K250A	DNA binding	$1.64x10^4$ +/- $6.5x10^2$	this paper
318-20A	DNA binding	$1.47x10^6$ +/- $6.1x10^4$	this paper
R370A	DNA binding	$1.2x10^6$ +/- $9.1x10^4$	this paper
D221S	Catalytic residue	ND	this paper
K246A	Catalytic residue	ND	this paper
D221N/E244Q	Catalytic residue	ND	this paper
D221N/E244A/ K246A (3X)	Catalytic residues	ND	this paper
Q129H	conserved	$1.0x10^3$ +/- 587	(11)
R139A	conserved	$1.1x10^3$ +/- 783	this paper

*previously characterized in vitro

Table of SOX mutants used in this study are listed by functional group.The indicated function of the mutated residue is noted and their catalytic efficiency on ssDNA is annotated, If relevant, with indication of the publication that characterizes their activity. ND is non detected and N/A is not applicable as these residues are involved in RNA processing.

Supplementary table 2

18S fwd	GTAACCCGTTGAACCCCATT
18S rev	CCATCCAATCGGTAGTAGCG
ACTB cds fwd	CATGTACGTTGCTATCCAGGC
ACTB cds rev	CTCCTTAATGTCACGCACGAT
GAPDH cds fwd	CTGGGCTACACTGAGCACC
GAPDH cds rev	AAGTGGTCGTTGAGGGCAATG
PP1A cds fwd	TCCTGGCATCTTGTCCATG
PP1A cds rev	CCATCCAACCACTCAGTCTTG
CTGF promoter fwd	CGAGGAATGTCCCTGTTTGT
CTGF promoter rev	ACTGGCTGTCTCCTCTCAGC
DDX6 cds fwd	CAGGAACATCGAAATCGTG
DDX6 cds rev	TCCAATACGATGGAGATAGG
PATI1 cds fwd	TCCTGCTCCCTATGGTGAGAG
PATI1 cds rev	CATGGCAGCAAGTGGACTACC
KSHV ORF59 promoter fwd	AATCCACAGGCATGATTGC
KSHV ORF59 promoter rev	CACACTTCCACCTCCCCTAA
KSHV ORF57 cds fwd	GGTGTGTCTGACGCCGTAAAG
KSHV ORF57 cds rev	CCTGTCCGTAAACACCTCCG
KSHV ORF59 cds fwd	GAAGGCAGTGGAGACGTTAGC
KSHV ORF59 cds rev	ACGGTGGAGGTGAGGTTGTC
KSHV K8.1 cds fwd	CCGTCGGTGTGTAGGGATAAAG
KSHV K8.1 cds rev	GTCGTTGTAGTGGTGGCAGAAA
KSHV ORF31 cds fwd	CAACATTCAAGAGGGCCAGT
KSHV ORF31 cds rev	ATGTCTGCAAATACCGCACA
KSHV ORF66 cds fwd	CCCATGCCACTTCTTCCTTA
KSHV ORF66 cds rev	CCACGTATGGGCAAAATACC
KSHV LANA cds fwd	TGGAGATGGGAGATGTAGGC
KSHV LANA cds rev	TTCGCCAACCGTAGTGTGTA

Table of primers used in this publication.
